# The Effect of Gastrostomy Placement on Gastric Function in Children: a Prospective Cohort Study

**DOI:** 10.1007/s11605-017-3376-3

**Published:** 2017-04-19

**Authors:** Josephine Franken, Femke A. Mauritz, Rebecca K. Stellato, David C. Van der Zee, Maud Y. A. Van Herwaarden-Lindeboom

**Affiliations:** 1 Department of Pediatric Surgery, Wilhelmina Children’s Hospital, University Medical Center Utrecht, Room KE.04.140.5, PO Box 85090, 3508 AB Utrecht, The Netherlands; 2 Department of Gastroenterology and Hepatology, Wilhelmina Children’s Hospital, Utrecht, The Netherlands; 3 Department of Biostatistics, Julius Center for Health Sciences and Primary Care, University Medical Center Utrecht, Utrecht, The Netherlands

**Keywords:** Gastrostomy, Gastric emptying, Motility, Gastroesophageal reflux

## Abstract

**Background:**

A gastrostomy placement is frequently performed in pediatric patients who require long-term enteral tube feeding. Unfortunately, postoperative complications such as leakage, feeding intolerance, and gastroesophageal reflux frequently occur. These complications may be due to postoperative gastric dysmotility. Our aim was to evaluate the effect of gastrostomy placement on gastric emptying in children.

**Methods:**

A prospective study was performed including 50 children undergoing laparoscopic gastrostomy. Before and 3 months after gastrostomy, assessment was performed using the ^13^C-octanoic acid breath test, 24-h pH monitoring, and reflux symptom questionnaires.

**Results:**

Gastric half-emptying time significantly increased from the 57th to the 79th percentile (*p* < 0.001) after gastrostomy (*p* < 0.001). Fifty percent of patients with normal preoperative gastric emptying develop delayed gastric emptying (DGE, *P* > 95) after gastrostomy (*p* = 0.01). Most patients (≥75%) with leakage and/or feeding intolerance after gastrostomy had DGE after operation. A decrease in gastric emptying was associated with an increase in esophageal acid exposure time (*r* = 0.375, *p* < 0.001).

**Conclusion:**

Gastrostomy placement in children causes a significant delay in gastric emptying. Postoperative DGE was associated with gastroesophageal reflux and was found in most patients with postoperative leakage and feeding intolerance. These negative physiologic effects should be taken into account when considering gastrostomy placement in children.

## Introduction

A gastrostomy placement (GP) is frequently performed in pediatric patients to provide prolonged enteral tube feeding. Although GP is a common procedure, the effects of the operation on gastric motility are unknown.

In the majority of patients, a GP is successful because, in time, sufficient caloric intake can be provided through the gastrostomy.^1^^,^ 2 However, in an estimated 15–25% of patients, a gastrostomy fails, leading to intolerance of feeding and leakage at the gastrostomy site.^3^^,^ 4 It is unclear whether these complications may be due to delayed gastric emptying (DGE) after the operation. Based on current evidence, it is unknown which patients are at risk of gastrostomy failure.^5^^,^ 6

Another widely discussed complication of GP is the development or deterioration of gastroesophageal reflux (GER). GER is frequently associated with abnormal gastric motility.^7^^,^ 8 DGE after GP may therefore be associated with postoperative GER, hence the importance of investigating GP and gastric motility.

In adults, the effect of a GP on gastric emptying (GE) has been investigated by two studies, detecting no significant changes in GE after operation.^9^^,^ 10 In children, only one retrospective study on GP and GE was performed, including 26 patients.^11^ This study was conducted with the ^13^C-octanoic acid gastric emptying breath test (^13C^GEBT) and detected no significant changes in GE after operation. This ^13C^GEBT is a reliable, safe, and noninvasive diagnostic method for GE in children.^12^ No prospective studies on GE before and after GP in children have been performed to date.

The aims of this study were to evaluate the effect of GP on GE in children using the noninvasive ^13C^GEBT and to identify parameters predictive of gastrostomy failure.

## Materials and Methods

### Study Design

A prospective, longitudinal cohort study including 50 pediatric patients was performed. Between May 2012 and April 2014, all children (aged 0–18 years) referred for GP to the Wilhelmina Children’s Hospital were considered for participation. Patients with a history of gastric surgery, with structural abnormalities of the stomach, or who were unable to undergo the assessment tests were excluded from the study.

### Ethical Approval and Trial Registration

The study was registered at the Dutch trial register before the start of the study (NTR3314, 29-02-2012). Ethical approval was obtained from the University Medical Center Utrecht Ethics Committee. Prior to initiating any study procedure, informed consent was obtained from the patients’ parents and the patients themselves (when 12 years or older and not neurologically impaired, NI).

### Surgical Procedure

In all children, a laparoscopic-assisted GP was performed under general anesthesia. All procedures were performed or supervised by an experienced pediatric surgeon. An infra-umbilical 6-mm trocar was introduced for the camera. The position of the gastrostomy was determined between the umbilicus and the costal margin. A small incision was made introducing a Babcock clamp to grasp the ventral wall of the gastric corpus under direct laparoscopic view. After pulling up the corpus, the gastric wall was sutured to the fascia of the abdominal wall with four interrupted sutures. After insufflation of the stomach, a needle was inserted through the stomach wall. Using the Seldinger technique, a peel-away dilator was placed followed by insertion of a gastrostomy tube. The gastrostomy balloon was inflated with sterile water.

On the first day after surgery, enteral feeding through the gastrostomy was initiated with half of the normal feeding regimen. On the second postoperative day, full enteral feeding was administered.

### Clinical Assessment

Patients underwent clinical assessment before and 3 months after GP. Clinical outcomes were analyzed with the ^13C^GEBT for GE analysis and with 24-h pH monitoring for GER analysis. Additionally, parents and children without NI over 12 years of age filled out a reflux-specific questionnaire.

### Gastric Emptying Test

GE was assessed with the ^13C^GEBT. For this ^13C^GEBT, the stable isotope ^13^C-labeled Na-octanoate is added to a solid or liquid test meal. This test has been proven to be a reliable, safe, and noninvasive diagnostic method for GE in children. In contrast to 99-Technetium scintigraphy, the former gold standard for GE, it offers normal values for children of all ages, both genders, and liquid and solid intake. Additionally, ^13C^GEBT does not involve radiation and is therefore suitable for large pediatric study populations.^13^ The intra-individual variability of the ^13C^GEBT has been studied in multiple studies. Hauser et al. found a coefficient of intrasubject variation of 12.5, which was comparable to the results of other studies. This variability is comparable with or even better than the variation reported by other techniques for GE measurement.^14^

Subjects fasted for at least 6 h before the study. In children >4 years of age, a solid ^13C^GEBT was performed with a 375-g pancake containing 45 mg of ^13^C-labeled Na-octanoate (a stable isotope). For younger children or children who were unable to eat the pancake within 15 min, 100 mg of ^13^C-labeled Na-octanoate was added to a liquid formula (infant formula, full cream milk, or chocolate milk). Breath samples were obtained in duplicate at 15-min intervals during the course of 4 h (for the liquid test, breath samples were obtained at 5-min intervals during the first 30 min). The ratio between ^12^CO_2_ and ^13^CO_2_ content in breath samples was analyzed with an isotope ratio mass spectrometer.

With this ^13C^GEBT, three parameters were calculated. Gastric half-emptying time (GE-T½) was defined as the time when the first half of the ^13^C-labeled substrate had been metabolized, that is, when the cumulative excretion of ^13^C in the breath was half the ingested amount. GE percentiles (*P*) were calculated according to the reference values obtained by M. van den Driessche et al.^15^ GE percentiles higher than 95 were considered delayed. The GE coefficient (GEC) reflects a global index for GE, influenced by both the rate of appearance and disappearance of ^13^C in breath.

### pH Monitoring

After 72-h cessation of anti-reflux medication, ambulatory 24-h pH monitoring was performed. A single-use multichannel intraluminal impedance pH catheter (Unisensor AG, Attikon, Switzerland) was calibrated in two different pH solutions and positioned transnasally into the distal esophagus with the probe located proximal to the lower esophageal sphincter. Correct catheter position was confirmed by fluoroscopy. For a 24-h period, acidity values were recorded in an ambulatory recorder. In a symptom diary, mealtimes, symptoms, body position (supine and upright), and other relevant events (e.g., correction of the catheter position) were documented. Automated analysis was performed with software designed for pH impedance analysis (Medical Measurement Systems). Pathological esophageal acid exposure was defined as total acid exposure time ≥6%, ≥9% in upright, and ≥3% in the supine body position.^16^

### Statistical Analysis

Continuous variables were expressed as the mean ± standard deviations (SD) for symmetric variables or as median with interquartile ranges (IQR) for skewed variables. Pre- and postoperative results were compared using the McNemar’s test for binary outcomes and the paired *t* test for continuous outcomes. Associations between categorical data were investigated with the chi-squared test or, in the case of small expected numbers, with the Fisher’s exact test. Correlations of continuous data were investigated with the Pearson’s correlation coefficient. Missing values were imputed using multiple imputation with 20 imputations. Descriptive statistics are reported for the original data; examination and testing of relations between variables was performed on the multiply imputed data.

To identify parameters predictive of gastrostomy failure, logistic regression analysis was performed. Potential risk factors were as follows: age, neurologic impairment, preoperative GE, acid exposure time, and symptomatic GER. Gastrostomy failure was defined as feeding intolerance or leakage at the gastrostomy site. Feeding intolerance was determined with the questionnaire that was filled out by parents scoring the vomiting symptoms of their child on a frequency scale (0–7 days a week) and a severity scale (0–7; Table 1). Patients with at least daily and moderately severe vomiting or at least weakly and severe vomiting (grade 2 or 3) were considered feeding-intolerant. Leakage at the gastrostomy site was determined by the indication for (re)admission or gastrojejunostomy placement.

**Table 1:** Scoring system that combines severity and frequency of symptoms of feeding intolerance

	Severe	Moderate	Mild	Absent
Daily	Grade 3	Grade 2	Grade 1	Grade 0
Weekly	Grade 2	Grade 1	Grade 1	Grade 0
Monthly	Grade 1	Grade 1	Grade 1	Grade 0
Infrequent	Grade 1	Grade 1	Grade 1	Grade 0

To identify parameters predictive of postoperative GE, multiple linear regression analysis was performed. Variables included in the analysis were the following: age, NI, preoperative GE, acid exposure time, and symptomatic GER. Statistical significance was defined by *p* values less than 0.05. All analyses were performed using SPSS 22.0 Statistical Package (IBM, USA).

## Results

A total of 50 patients were included, with a median age of 3.4 years (1.4–5.6). Indication for gastrostomy was insufficient oral caloric intake in 47 patients. The remaining three patients received a gastrostomy for administering laxatives in chronic obstipation. The main underlying pathologies were neurological disorder (68%) and cystic fibrosis (8%). Patient characteristics are described in Table 2.

**Table 2:** Patient characteristics

Demographics	
Total number of patients	50
Male gender, *n* (%)	29 (58)
Age at time of operation (years), median (IQR)	3.4 (1.4–5.6)
Follow-up time (months), median (IQR)	4.6 (3.7–5.6)
Main underlying disorder, *n* (%)	
Neurologic impairment	34 (68)
Cystic fibrosis	4 (8)
Chronic obstipation	3 (6)
Failure to thrive with unknown diagnosis	3 (6)
Congenital cardiac disease	2 (4)
Metabolic disorder	2 (4)
Pulmonary disease	1 (2)
Short bowel syndrome	1 (2)

*n* number, *IQR* interquartile range

**Fig. 1:**
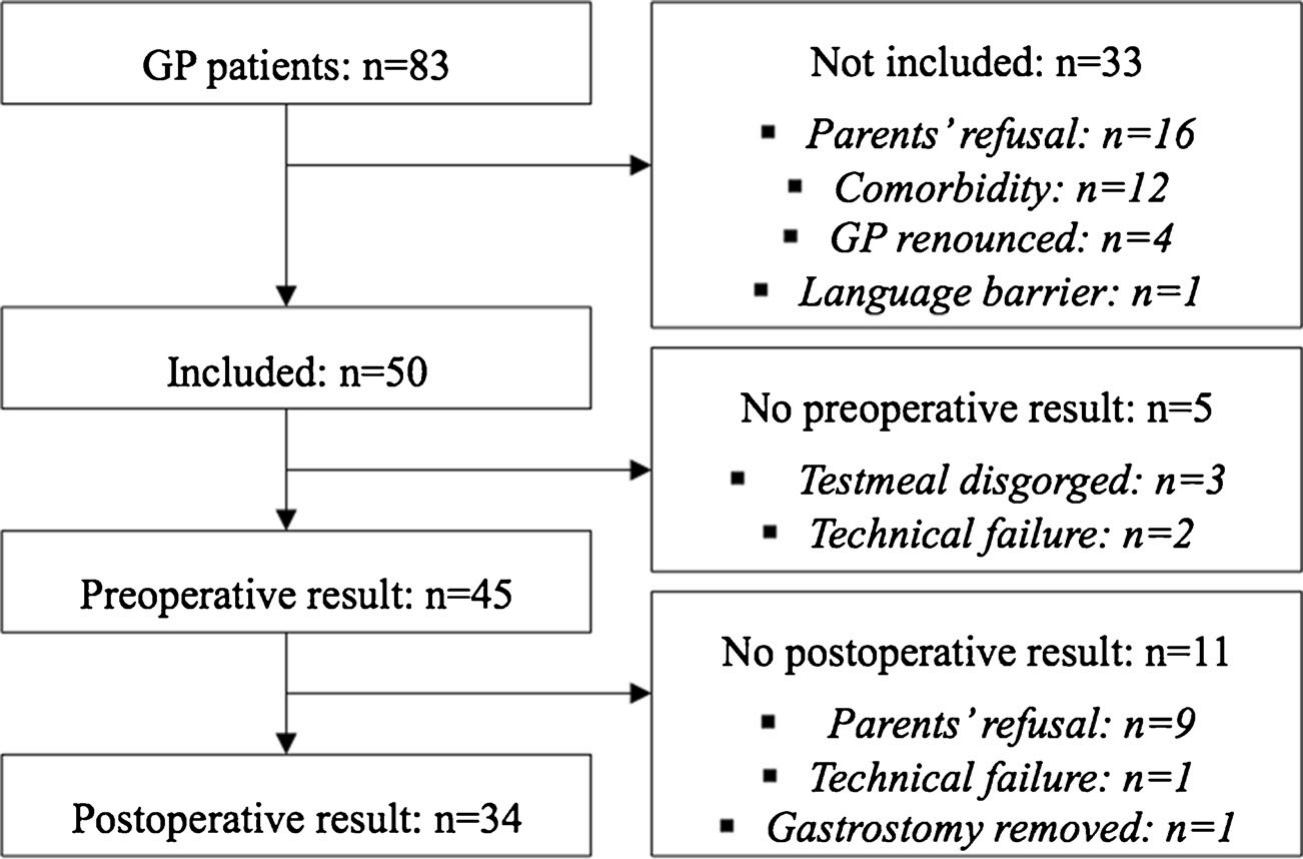
Flowchart of patient inclusion. *GP* gastrostomy placement, *n* number

Preoperative ^13C^GEBT was performed successfully in 45 patients. In 34 of these patients, ^13C^GEBT was also completed successfully after operation (Fig. 1). In nine patients, ^13C^GEBT could not be repeated due to parents’ refusal. These parents considered the postoperative test as too much of a burden. In one patient, the gastrostomy was removed 2 months after gastrostomy at the request of parents because of repetitive leakage at the gastrostomy site. One postoperative test could not be completed due to technical failure. Liquid ^13C^GEBT was performed in 40 (89%) of the preoperative tests and in 32 (94%) of the postoperative tests; the remaining tests were performed with solid intake.

Twenty-four-hour pH monitoring was performed in all patients before operation and repeated after gastrostomy in 28 patients (56%). All parents filled out the reflux-specific questionnaires.

### Symptoms

Almost all patients (49/50) still received gastrostomy feeding at 3 months follow-up. The majority of patients (73%) were able to receive enteral feeding in boluses; the remaining 27% were dependent on continuous drip feeding (either solely overnight or 24 h per day). Seventy percent of patients with gastrostomy feeding received additional oral feeding; the other 30% of patients was entirely dependent on feeding through the gastrostomy tube. Gastrostomy failure, caused by leakage (*n* = 6) and/or feeding intolerance (*n* = 8), occurred in ten patients (20%) after GP.

### Gastroesophageal Reflux

After GP, the acid exposure time remained similar (preoperative = 6.1% (2.7–16.0) and postoperative = 6.1% (2.8–12.1), *p* = 0.866, *n* = 28). Four patients (14%) developed pathological GER after GP, whereas pathological GER disappeared in the same number of patients. GER symptoms were present in a comparable number of patients before (44%) and after GP (39%, McNemar *p* = 0.73).

### Gastric Emptying

After gastrostomy, the GE rate significantly decreased compared to preoperative GE rate: the GE percentile and the GE-T½ both significantly increased (*p* < 0.001 and *p* = 0.03, respectively) and the GEC decreased significantly (*p* < 0.001; Table 3).

**Table 3:** Gastric emptying before and after GP (*n* = 50)

	Before GP	After GP	*p* value^a^
GE percentile (SD)	57 (±36.6)	79 (±30.7)	<0.001
GEC (SD)	3.8 (±0.90)	3.5 (±0.86)	<0.001
GE-T½ (min, IQR)	45 (24–70)	71 (39–94)	0.03

*GE* gastric emptying, *SD* standard deviation, *GEC* gastric evaluation coefficient, *GE*-*T*½ gastric half-emptying time, *IQR* interquartile range

^a^Paired *t* test

**Fig. 2:**
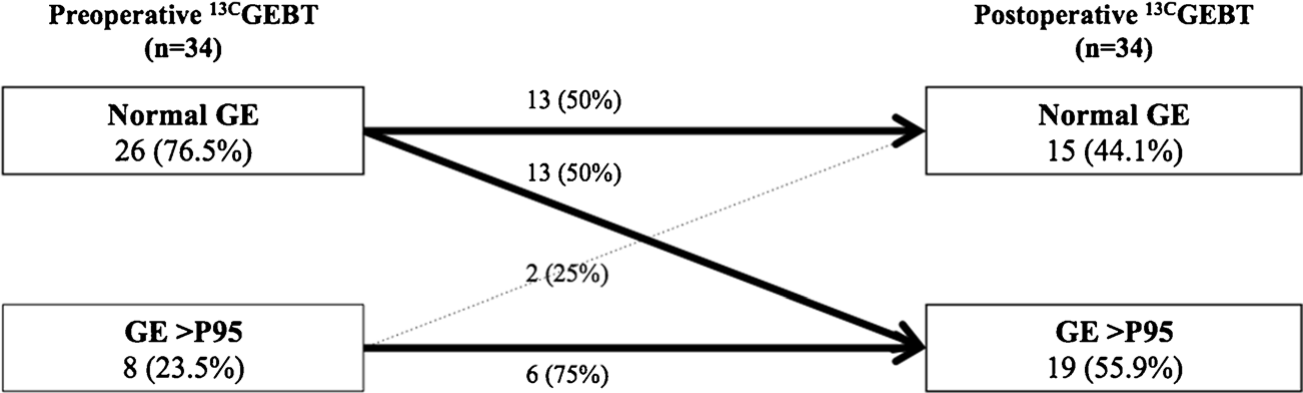
Gastric emptying before and after GP (*n* = 34). *GP* gastrostomy placement, ^13C^ *GEBT 13*-*C* octanoic acid gastric emptying breath test, *GE* gastric emptying, *P* percentile

In 26 patients (76.5%), GE was normal before operation. After gastrostomy, 50% of these 26 patients developed DGE (McNemar *p* = 0.01; Fig. 2). Before gastrostomy, DGE (*P* > 95) was present in eight patients. After operation, this number increased to 19 patients (56%, McNemar *p* = 0.01).

After dividing the patients into two subgroups: patients with NI and neurologically normal (NN) patients, sub-analysis showed that NI patients had a higher GE percentile before operation (P62 (±36.5) vs. P57 (±36.6); Table 4). The GE percentile in NI patients significantly increased to P84 (±27.9) after operation (*p* < 0.001), a similar increase compared to the NN patients.

**Table 4:** Sub-analysis of NI patients (*n* = 34)

	Before GP	After GP	*p* value^a^
GE percentile (SD)	62 (±36.5)	84 (±27.9)	0.004
GEC (SD)	3.9 (±0.95)	3.5 (±0.69)	0.004
GE-T½ (min, IQR)	44 (27–64)	66 (49–93)	0.03

*GE* gastric emptying, *SD* standard deviation, *GEC* gastric evaluation coefficient, *GE*-*T*½ gastric half-emptying time, *IQR* interquartile range

^a^Paired *t* test

### Sequelae of DGE

A ^13C^GEBT was completed in four out of six patients with leakage after gastrostomy, all showing DGE (100%, Fisher’s exact *p* = 0.11). In patients with feeding intolerance, postoperative ^13C^GEBT showed DGE in six out of eight patients (75%, Fisher’s exact *p* = 0.25).

A positive correlation was found between GE-T½ and esophageal acid exposure time, both before (*r* = 0.28, *p* < 0.001) and after GP (*r* = 0.46, *p* < 0.001, *n* = 28). Increased acid exposure time after GP was correlated with increased GE-T½ (*r* = 0.375, *p* < 0.001). No significant correlation was found between postoperative GE-T½ and GER symptoms (*r* = 0.016, *p* = 0.624).

### Risk Factors

In univariable analysis of failure after GP, none of the characteristics examined were statistically significant predictors (Table 5). In multivariable analysis of postoperative GE, preoperative GE was a positive predictor (*B* = 0.3, 95% CI = 0.04–0.6). Age and NI were not identified as predictors of postoperative GE (Table 6).

**Table 5:** Predictors of gastrostomy failure: univariable analysis (*n* = 50)

Predictors (preoperative)	*p* value	Predictive value (odds)	95% CI
Age (years)	0.11	0.84	0.67–1.05
Acid exposure time (%/24 h)	0.17	0.93	0.85–1.03
Neurologic impairment (yes/no)	0.18	0.44	0.11–1.54
GE (percentile)	0.27	0.99	0.97–1.01
Symptomatic GER (GSQ)	0.50	1.01	0.98–1.04

*GE* gastric emptying, *CI* confidence interval, *GER* gastroesophageal reflux, *GSQ* gastroesophageal reflux symptom questionnaire

**Table 6:** Predictors of postoperative GE percentile: multivariable analysis (*n* = 50)

Predictors	*p* value	Predictive value (*B*)	95% CI
Preoperative GE (percentile)	0.03	+0.3	+0.04 to +0.6
Neurologic impairment (yes/no)	0.32	+9.8	−9.5 to +29.2
Age (years)	0.71	+0.5	−2.0 to +3.0

*GE* gastric emptying, *CI* confidence interval

## Discussion

In this prospective pediatric study, we found that gastrostomy placement causes a significant decrease in GE rate. Fifty percent of patients with a normal preoperative GE develop DGE after GP.

This is the first prospective study on GE before and after GP involving pediatric patients. Only one previous study was published on this subject.^11^ This was a retrospective study including 26 NI children undergoing laparoscopic GP. In contrast to our study, the authors reported no significant changes in GE after operation. This difference in outcome may partly be explained by a lack of statistical power in that study (*n* = 26).

In adults, two studies have been performed on GE after GP, showing no significant changes. The first study reported a non-significant delay in GE-T½.^9^ The fact that this delay was not significant may have been due to the small number of participants (*n* = 11). The second study found that GE was unaffected after GP.^10^ GE testing in this study was, however, conducted with the paracetamol absorption test, i.e., plasma concentrations of paracetamol at 45 min after drug administration. This diagnostic technique still needs further standardization before it can reliably be used for research purposes.^17^

Furthermore, the results of adult studies cannot be translated to the pediatric population, mainly because indications for GP differ. In the adult population, gastrostomy placements are primarily performed in patients with head and neck malignancies, whereas in the pediatric population, patients often suffer from profound neurological impairment.^18^ Generalized gastrointestinal dysmotility is frequently encountered in these patients,^19^^,^ 20 and GI motility changes after GP may consequently differ. Well-designed studies confined to the pediatric population are therefore necessary.

The cause for the delay in GE is not evident. A previous study reported that myoelectrical activity, relevant to gastric motor function, was unaffected after GP.^10^ Slow fundic contractions are believed to transfer gastric contents from the fundus to the antrum for trituration and subsequent GE. These contractions might be affected by gastrostomy placement in the gastric body.^21^ To clarify the cause for delay in GE, motility tests such as three-dimensional ultrasonography or dynamic contrast-enhanced magnetic resonance imaging of the stomach may be useful.

The effect of a GP on GER has been a matter of profound debate. A systematic review showed that evidence has been inconsistent and of insufficient methodological quality.^22^ In our study, the total acid exposure time did not change significantly, supporting previous findings that GER generally does not worsen after GP.^3^^,^ 22 ^,^ 23

DGE is associated with GER, based on the positive correlation between GE-T½ and acid exposure time, both before and after gastrostomy. This is in line with previous studies reporting on this pathophysiologic relationship.^24^^,^ 25 Furthermore, we found that changes in acid exposure time after GP were correlated to changes in GE. Thus, development or worsening of GER after GP, which was frequently reported by other studies,^26^^,^ 27 seems to be influenced by a delay in GE.^28^ Other factors may also play a role in the pathogenesis of GER after gastrostomy, e.g., changes in lower esophageal sphincter pressure^29^ or the presence of esophageal hiatus hernia.^10^

Postoperative DGE may stimulate problems such as leakage and intolerance of feeding. No previous studies have reported on this causality. According to our findings, most patients with complications of leakage and feeding intolerance were found to have postoperative DGE. Analysis in larger study populations is required to provide more certainty on the causality between DGE and gastrostomy failure.

Children undergo GP for a wide variety of indications. The majority of children in our cohort suffered from NI (68%). It is well known that these children often suffer from generalized gastrointestinal dysmotility. This may have resulted in slower GE in NI children compared to NN children in our cohort. For this reason, we performed a sub-analysis of NI children alone. It indeed showed a higher GE percentile before operation. However, after operation, the delay in GE was similar (both NI and NN made an increase of 22 percentile points).

Unfortunately, we were unable to identify preoperative predictors of gastrostomy failure. The number of patients with gastrostomy failure (*n* = 12) was too low to perform multivariable analysis. The occurrence of gastrostomy failure might be multifactorial or dependent on factors not included in our univariate analysis.

To our knowledge, no previous study has identified predictors of gastrostomy failure in children. However, two studies attempted to identify predictors of all minor gastrostomy-related complications (including, e.g., hypergranulation and stomal infection). The first study identified no significant predictors.^6^ The other reported a higher frequency of complications in patients with cardiac malformations (*n* = 17).^5^ Future research dedicated to this subject may provide us with more insight into risk factors for complications after GP.

A limitation of this study was that 11 postoperative ^13C^GEBTs were missing. In order to maintain adequate statistical power, we performed multiple imputation analysis on ^13C^GEBT results. Analysis of the imputed data yielded results similar to those of the original data. This suggests a random missing of the postoperative ^13C^GEBT, therefore making a bias on the effect sizes less probable.

## Conclusion

In conclusion, this is the first study that demonstrates a delay in GE after a GP in children. Patients with a normal preoperative GE have a 50% chance of developing DGE after GP. DGE after GP is associated with GER and is found in most patients with postoperative leakage and feeding intolerance. Although gastrostomy failure could not be predicted with preoperative data, the negative effect of GP on GE and its possible consequences should be taken into account when this operation is considered in pediatric patients.
